# Childhood Trauma in Persons With Schizophrenia and a History of Interpersonal Violence

**DOI:** 10.3389/fpsyt.2020.00383

**Published:** 2020-05-05

**Authors:** Guttorm Breivik Storvestre, Arvid Jensen, Espen Bjerke, Natalia Tesli, Cato Rosaeg, Christine Friestad, Ole Andreas Andreassen, Ingrid Melle, Unn Kristin Haukvik

**Affiliations:** ^1^Department of Psychiatry, Ostfold Hospital Trust, Grålum, Norway; ^2^NORMENT, Norwegian Centre for Mental Disorders Research, Oslo University Hospital, Oslo, Norway; ^3^Centre of Research and Education in Forensic Psychiatry, Oslo University Hospital, Oslo, Norway; ^4^Department of Research, University College of Norwegian Correctional Service, Lillestrøm, Norway; ^5^NORMENT, Norwegian Centre for Mental Disorders Research, Institute of Clinical Medicine, University of Oslo, Oslo, Norway; ^6^Department of Adult Psychiatry, Institute of Clinical Medicine, University of Oslo, Oslo, Norway

**Keywords:** emotional neglect, physical neglect, physical abuse, sexual abuse, forensic psychiatry, psychosis

## Abstract

**Background:**

Childhood trauma is a risk factor for psychosis as well for violent behavior and offending later in life. Childhood trauma comprises subdomains of abuse and neglect that may be differently related to later violence among patients with schizophrenia. The aim of this study was to map the subdomains of childhood trauma associated with violent offending in schizophrenia.

**Methods:**

Information on childhood trauma from predominantly male patients with a DSM-IV diagnosis of schizophrenia and a history of violent offending (interpersonal violence) (SCZ-V, n = 19), schizophrenia patients without a history of violence (SCZ-NV, n = 34), and healthy controls (HC, n = 66) was obtained with the Childhood Trauma Questionnaire (CTQ). Differences between groups in total maltreatment scores and the five subdomains including physical, emotional, and sexual abuse, as well as physical and emotional neglect were analyzed.

**Results:**

SCZ-V had the highest median CTQ scores for all sub-domains. SCZ-V reported significantly higher total CTQ scores than SCZ-NV and HC. SCZ-V had significantly higher scores than HC on all subdomains, and significantly higher than SCZ-NV on physical and emotional neglect. SCZ-NV had higher scores on all domains except sexual abuse compared to HC.

**Conclusion:**

SCZ-V patients had higher exposure to childhood trauma than SCZ-NV, and both schizophrenia groups had higher exposure than HC. The results suggest that childhood physical and emotional neglect may be of specific importance to later violence in schizophrenia.

## Introduction

Schizophrenia patients are at a small but significantly increased risk of engaging in violent behavior compared to the general population (1–3). Such acts may attract substantial negative media attention, and thus add to the burden of an already stigmatized group of patients. The exact mechanisms behind the increased violence risk among persons with psychosis have not been determined, but violence risk has been shown to be influenced by psychosis symptoms such as delusions, paranoid ideation (2) and hallucinations, as well as by reduced impulse control, sometimes further increased by drug or alcohol use (4–6).

Clinical and epidemiological studies have shown that individuals who have experienced childhood maltreatment/trauma have an increased risk for violent behavior later in life (7, 8). Childhood maltreatment is also associated with an increased risk of developing psychosis (9–11) compared to the general population, and most probably with an increased risk of violent behavior among patients with psychosis (12–15).

Despite higher prevalence of childhood trauma among people suffering from psychotic disorders, and the known increased risk of violent behavior in the same patient group, few studies have investigated the association between childhood trauma and violent behavior in patients with psychotic disorders. A systematic review and meta-analysis published in 2017 (16) found only five English language studies of childhood maltreatment and violent behavior during psychosis in adults (14, 17–20). The meta-analysis showed that psychosis patients with a history of childhood trauma were approximately twice as likely to commit interpersonal violence compared to psychosis patients without a childhood trauma history (16). The results are in line with findings from a forensic psychiatry patient cohort (> 90% had disorder in the schizophrenia spectrum) where childhood trauma and insecure attachment predicted later violence risk (13). Studies of non-psychotic subject samples have corroborated the association between exposure to physical abuse in childhood and later perpetration of interpersonal violence (21–23). To reduce violence risk among psychosis patients, there is a need to clarify specific patterns of childhood trauma associated with violence later in life.

One of the most widely used instruments to measure childhood trauma is the validated self-report “Childhood Trauma Questionnaire” (CTQ). The CTQ consists of five subscales, differentiating between types of trauma: physical, emotional, or sexual abuse, and physical or emotional neglect. Bosqui and colleagues identified an association between violence risk and childhood trauma in general, and between violence and the subdomains of physical abuse and neglect, and sexual abuse in particular, among psychosis patients in general; when controlling for possible under-reporting, sexual abuse remained significant (14). Also using the CTQ, a study by Bennouna-Greene and colleagues among schizophrenia patients with a history of violent offending reported a high frequency of childhood trauma in general and a high prevalence of physical abuse (24) in particular. However, this study did not include a comparison group of non-violent schizophrenia patients nor healthy controls. We thus do not know to what extent the association is mediated by the presence of psychosis, or if there are specific associations to violence within the psychosis patient group.

The aim of the current study was to explore the relationship between exposure to different types of childhood trauma and violent behavior in a sample of forensic patients with schizophrenia and a history of violent offending (SCZ-V), compared to schizophrenia patients without a history of violence (SCZ-NV) and to healthy controls (HC). In particular, we investigated the differences between the groups regarding both the level of total childhood trauma as well as the subdomains. Based on previous findings, we hypothesized that SCZ-V had been exposed to a higher total load of childhood trauma, including both abuse and neglect compared both to SCZ-NV and HC, but with a specific increase in the subdomain related to physical abuse.

## Methods

The subject sample (n = 119) consisted of patients with schizophrenia and a history of severe violence toward others (murder, attempted murder, or severe violence toward other persons) (SCZ-V, n = 19) recruited from six high security forensic psychiatry wards in Norway, at Ostfold Hospital and Oslo University Hospital. Age and sex matched schizophrenia patients without a history of violence (SCZ-NV, n = 34), and healthy controls (HC, n = 66) were recruited from the on-going multi-centre Thematically Organized Psychosis (TOP) Study at the University of Oslo. SCZ-NV were recruited from four major psychiatric hospitals and their out-patient clinics, that together cover most of the population in the city of Oslo. The healthy control subjects were randomly selected from the national population register. The subject sample has previously been included in two imaging studies (25, 26). For all groups, exclusion criteria were age under 18 or over 70 years and lack of Norwegian language knowledge sufficient to understand the study procedures and information to provide written consent. Participant inclusion took place between 2014 and 2016.

The study was approved by the Regional Committee for Medical Research Ethics and the Norwegian Data Inspectorate, and it was conducted in accordance with the Helsinki declaration. After presenting a complete description of the study to the subjects, written informed consent was obtained from all participating subjects.

### Clinical Assessment

All patients were thoroughly assessed by specially trained psychologists and physicians. They were referred to the study by their treating psychologist or psychiatrist who performed the first screening for eligibility, which included safety evaluations for the SCZ-V group. From the SCZ-V group, two patients dropped out after the start of inclusion procedures, one because of hospital transfer and the other because of change of mind. Clinical diagnoses were confirmed using the Structured Clinical Interview for DSM-IV axis 1 disorders (SCID-I) module A–E (27) with intra-rater agreement of 82%, kappa = 0.77 (95% CI 0.60–0.94). Psychosocial functioning was assessed with the Global Assessment of Functioning (GAF) scale, split version (28). Ongoing psychotic symptoms were rated by the use of the Positive and Negative Syndrome Scale (PANSS) (29), with intra-class correlation (1.1) of 0.73. G.B.S, U.K.H and C.R. performed the clinical inclusion of the SCZ-V at the forensic security wards to minimize the risk of violent episodes during study procedures. The nurses at the forensic psychiatry wards or the treating psychologists or psychiatrists were present during parts of the patient inclusion. To reduce stress for participants in the SCZ-V group, in a few cases, the DSM-IV diagnoses were obtained from their medical records and forensic reports and not from interviews. For patients in the SCZ-NV group, the medical files and PANSS-scores were examined to ensure absence of previous or current violence (i.e., murder, attempted murder, or criminal assault). The healthy controls were assessed with the PRIME-MD, which is a screening instrument designed for general practice, by trained psychologist to ensure that no severe mental disorder was present.

### Childhood Trauma Questionnaire

A history of childhood trauma was assessed according to the CTQ, a validated self-report retrospective instrument administered to register and quantify experiences of childhood maltreatment in the person’s home (30). We used the 28 items CTQ (revised) (31). Norwegian version (32), in which the five clinical subscales are measured by five items each. The scale includes three items representing a denial/minimization subscale, to indicate potential underreporting of maltreatment. Each item is scored on a five-point Likert scale (1 = ”never” to 5 = ”very often”), making the sum of the five subscales range from 5 to 25.

### Statistical Analyses

All statistical analyses were performed using the statistical package SPSS version 26 (IBM, SPSS Inc., Armonk, New York, USA). Demographic and clinical variables were investigated by analysis of variance (ANOVA), independent samples T-test, and Chi-Square analysis between diagnostic groups. All tests were two-tailed with an alpha threshold of 0.05.

**Figure 1:**
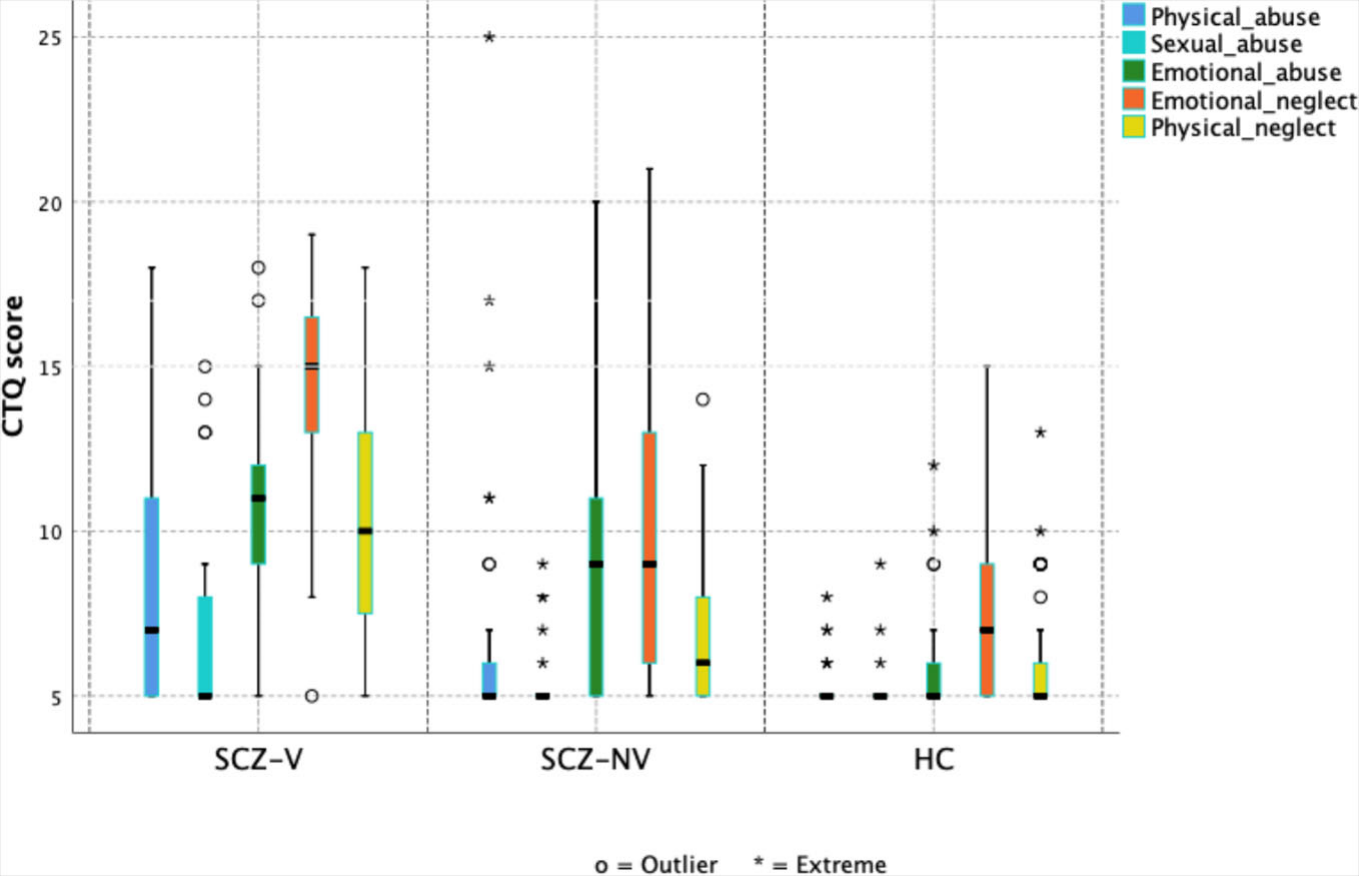
Box plots showing the distribution of childhood trauma (CTQ scores) in total and across subdomains, stratified by diagnostic group. SCZ-V, Schizophrenia with a history of violence (n = 19); SCZ-NV, Schizophrenia without a history of violence (n = 34), HC, Healthy controls (n = 66).

Group differences in total maltreatment and subscale scores (physical, sexual, and emotional abuse, and physical and emotional neglect), were analyzed with the Kruskal-Wallis H test, because of the non-normal distribution of scores (**Table 2**, **Figure 1**). *Post hoc* pairwise group comparisons were performed with the Mann-Whitney U test, with Bonferroni adjustment for the three contrasts (SCZ-V vs. SCZ-NV, SCZ-V vs. HC, and SCZ-NV vs. HC).

To evaluate the effect of gender we performed a *post hoc* multiple linear regression analysis with total CTQ (transformed to its natural logarithm) as the dependent, and gender, case vs. control status and violence vs. non-violence as independents. Residual plots were examined, in addition to the effect of outliers and of influential observations.

## Results

### Demographic and Clinical Variables

The SCZ-V group had fewer years of education compared to SCZ-NV and HC, while the SCZ-NV group was younger than the SCZ-V and HC groups. The two patient groups differed in age at first psychosis onset as well as age at first admission, with the SCZ-V group being younger for both. There were no significant group differences in the other clinical and demographic variables (**Table 1**).

**Table 1:** Demographic and clinical characteristics.

	Schizophrenia *n* = 34		Violent Schizophrenia *n* = 19		Control subjects *n* = 66		Statistics *p*-value
	*Number*	*%*	*Number*	*%*	*Number*	*%*	*Chi-square*
**Sex (m/f)**	33/1	97/3	18/1	95/5	64/2	97/3	NS
	***Mean (SD)***	***Range***	***Mean (SD)***	***Range***	***Mean (SD)***	***Range***	***ANOVA***
**Age (years)**	27.2 (6.7)	19–48	33.2 (9.4)	19-54	32.3 (7.1)	19–46	0.002
**Years of education** *(n = 34/16/66)*	13.3 (2.9)	4.5–20	9.9 (1.8)	6-15	14.4 (2.9)	12–25	0.000
	***Number***	***%***	***Number***	***%***	***Number***	***%***	***Chi-square***
**Cannabis, last 2 years (no/yes)**	14/20	41/59	11/7	61/39			NS
	***Mean (SD)***	***Range***	***Mean (SD)***	***Range***	***Mean (SD)***	***Range***	***t-test***
**Alcohol last 2** **years (units)** *(n = 31/16/-)*	428.0 (778.3)	0–4160	216.4 (539.6)	0–1872			NS
**GAF symptom** *(n = 34/17/-)*	47.4 (16.3)	21–91	41.8 (13.9)	28–73			NS
**GAF function** *(n = 34/17/-)*	46.9 (14.9)	21–85	39.1 (15.5)	20–78			NS
**PANSS positive** *(n = 34/16/-)*	14.6 (5.7)	7–32	16.4 (7.5)	7–28			NS
**PANSS negative** *(n = 34/16/-)*	17.4 (6.7)	7–43	18.6 (5.8)	8–27			NS
**PANSS general** *(n = 34/16/-)*	32.8 (9.7)	17–69	30.2 (10.9)	18–49			NS
**Age at psychosis onset** (n = 33/17/-)	23.1 (4.5)	16–38	18.4 (5.9)	10–30			0.003
**Age at first psychosis admission** *(n = 31/14/-)*	24.8 (4.9)	18–41	20.0 (5.2)	10–29			0.005

m/f, male/female; SD, standard deviation; GAF, Global Assessment of Function split version; PANSS, Positive and Negative Syndrome Scale; ns, non-significant.

### Childhood Trauma

SCZ-V and SCZ-NV reported higher median total CTQ scores than HC (**Table 2**, **Figure 1**.) A Kruskal-Wallis H test showed a significant group difference (χ^2^(2) = 40.8, p = 1.4 × 10^−9^), with mean rank score of 96.5 for SCZ-V, 71.8 for SCZ-NV, and 43.4 for HC (**Table 2**). Mann-Whitney pairwise comparisons showed significant between-group differences, with higher scores for SCZ-V than SCZ-NV (U = 161.5, p =.009), SCZ-V than HC (U = 94, p = 5.1 × 10^−8^), and SCZ-NV than HC (U = 559.5, p =.0001) (**Table 3**).

**Table 2:** Childhood trauma in patients with (SCZ-V) and without (SCZ-NV) a history of violence, and healthy controls (HC).

	SCZ-V		SCZ-NV		HC		Group comparison	
	*n = 19*		*n = 34*		*n = 66*		(Kruskal-Wallis H test)	
CTQ	**Median (Q)**	**Range**	**Median (Q)**	**Range**	**Median (Q)**	**Range**	Mean rank (SCZ-V, SCZ-NV, HC)	χ^2^(2), p-value
Total trauma	55 (40, 59)	25–77	33.5 (30, 44)	25–76	29 (26, 31.3)	25–42	96.5, 71.8, 43.4	40.8, 1.4 × 10^−9^
**Subdomains:**								
Physical abuse	7 (5, 11)	5–18	5 (5, 6.3)	5–25	5 (5, 5)	5–8	86.6, 66.8, 48.8	32.1, 1.4 × 10^−9^
Sexual abuse	5 (5, 9)	5–15	5 (5, 5)	5–9	5 (5, 5)	5–9	75.3, 61.0, 55.1	15.4, 0.0005
Emotional abuse	11 (9, 12)	5–18	9 (5, 11)	5–20	5 (5, 6.3)	5–12	89.6, 74.4, 44.0	37.2, 8.5 × 10^−9^
Physical neglect	10 (7, 13)	5–18	6 (5, 8.3)	5–14	5 (5, 6)	5–13	95.1, 64.7, 47.5	34.8, 2.8 × 10^−8^
Emotional neglect	15 (12, 17)	5–19	9 (6, 13)	5–21	7 (5, 9)	5–15	95.4, 66.3, 46.6	31.6, 1.3 × 10^−7^

SCZ-V, Schizophrenia with a history of violent offending; SCZ-NV, Schizophrenia without a history of violent offending; HC, Healthy controls; CTQ, Childhood Trauma Questionnaire short-form; Q, 25% percentile, 75% percentile.

**Table 3:** Pairwise comparisons of childhood trauma scores between patients with (SCZ-V) and without (SCZ-NV) a history of violence, and healthy controls (HC).

	SCZ-V vs. SCZ-NV		SCZ-V vs. HC		SCZ-NV vs. HC	
CTQ	**Mann Whitney U**	p-value*	**Mann Whitney U**	p-value*	**Mann Whitney U**	p-value*
Total trauma	161.5	.009	94	5.1 × 10^−8^	559.5	.0001
**Subdomains:**						
Physical abuse	214	.09	231	3.0 × 10^−8^	780.5	.0006
Sexual abuse	241	.117	417.5	.0003	1007	.225
Emotional abuse	223	.183	164.5	5.4 × 10^−7^	531	.00002
Physical neglect	136	.001	146.5	3.9 × 10^−8^	775	.012
Emotional neglect	154.5	.006	123.5	2.5 × 10^−7^	739	.015

SCZ-V, Schizophrenia with a history of violent offending n = 19; SCZ-NV, Schizophrenia without a history of violent offending n = 34; HC, Healthy controls n = 66; CTQ, Childhood Trauma Questionnaire short-form.

*Bonferroni corrected p-value.

Kruskal-Wallis H tests showed significant group differences for all five CTQ-subdomains, physical abuse (χ^2^(2) = 32.1, p = 1.4 × 10^−9^), sexual abuse (χ^2^(2) = 15.4, p = 0.0005), emotional abuse (χ^2^(2) = 37.2, p = 8.5 × 10^−9^), physical neglect (χ^2^(2) = 34.8, p = 2.8 × 10^−8^), and emotional neglect (χ^2^(2) = 31.6, p = 1.3 × 10^−7^) (**Table 2**, **Figure 1**). Mann-Whitney pairwise comparisons showed significantly higher scores for SCZ-V compared to SCZ-NV for physical neglect (p =.001) and emotional neglect (p =.006) (**Table 3**). SCZ-V had significantly lower scores on all sub-domains compared to HC (**Table 3**). SCZ-NV had significantly lower scores on all sub-domains, except sexual abuse, compared to HC (**Table 3**).

The post doc analysis investigating the influence of gender did not find any significant gender effects (p = 0.78).

## Discussion

The main findings in this study were statistically significant differences in total childhood trauma exposure between all diagnostic groups. The SCZ-V group had higher CTQ scores than the SCZ-NV group, and both patient groups had higher scores than the HC group. On the subdomain level, we found a similar pattern, but, noteworthy, a higher exposure to physical and emotional neglect among SCZ-V than SCZ-NV.

### Total Childhood Trauma Exposure

Since childhood trauma is a risk factor both for schizophrenia and violence, we expected significantly higher CTQ scores in both SCZ-V and SCZ-NV compared to HC. Our predominantly male SCZ-NV group had a median score of 33.5 for total trauma, which is in line with the results from previous studies of childhood trauma and specifically CTQ-scores on mixed gender schizophrenia samples (33–36). Moreover, our SCZ-V group had a median score of total trauma of 55, which was significantly higher than our two control groups and clearly higher than the mean or median values found in most general schizophrenia studies as cited above. As such, our finding of higher total childhood trauma experienced by SCZ-V supports the first part of our hypothesis; the SCZ-V patients had been exposed to more childhood trauma compared to SCZ-NV and HC.

Childhood trauma is not only related to violence in schizophrenia but also with poorer outcomes (36). The specific mechanisms underpinning this association are not known. However, overwhelming stress in childhood has been associated with changes in brain development in patients who later develop psychotic disorders (37). Indeed, psychosis patients with a history of childhood trauma have shown structural and functional brain abnormalities as well as impaired cognitive functioning (38), with widespread white matter microstructure abnormalities (decreased fractional anisotropy) (39), decreased connectivity between the posterior cingulate cortex and the amygdala (40), and altered activation in parietal and visual areas during a working memory task (41) [see (42) for review]. Interestingly, the anterior cingulate cortex, a region which is involved in higher cognitive processes such as moral reasoning, attention, and motivation, is smaller in violent offenders with schizophrenia or antisocial personality disorder who have been exposed to childhood trauma (17). Furthermore, Aas et al. reported that childhood trauma was associated with recognition of facial expressions; patients with schizophrenia and childhood trauma history evaluated negative faces as more negative, and positive faces as less positive than schizophrenia patients without exposure to childhood trauma (43). We have previously reported cortical folding alterations in the visual association and orbitofrontal cortex among in an MRI study comprising participants from the current study (Storvestre et al., 2019). Taken together, these findings may suggest that childhood trauma could be associated with social cognitive abilities and attention, as well as brain circuits relevant to aggressive behavior and may point toward some of the mechanisms underpinning the association between childhood trauma and later violence as observed in the current study.

### Specific Childhood Trauma Subdomain Exposure

On the subdomain level, SCZ-V reported exposure to more physical and emotional neglect than SCZ-NV, but no difference in the exposure to sexual, emotional, or physical abuse. The lack of difference in exposure to physical abuse is surprising since previous studies have reported a specific association between being exposed to physical abuse and later violent offending or risk in both psychosis patients (14, 24) and non-psychotic persons (21–23). However, a large-scale study comprising almost 3,000 males showed that experiencing childhood physical abuse was specifically associated with later intimate partner violence, whereas neglect was significantly associated with violence toward strangers, both mediated by anti-social personality disorder but not psychosis (44). In the same study, psychosis (together with anti-social personality disorder) mediated later violence risk associated with having witnessed domestic violence in childhood (44). A large epidemiological study comprising almost 30,000 respondents identified a relationship between experiencing harsh physical punishment in childhood with increased odds for exposure to childhood emotional, sexual abuse, and physical abuse, as well as physical and emotional neglect, after controlling for confounding sociodemographic factors (45). As such, the association between specific subtypes of childhood trauma and later violence risk may not be so clear-cut as previously thought, but rather affected by types of violent offence and confounded by the simultaneous presence of other types of childhood maltreatment. This could explain the discrepancy between the current study and previous findings.

The association between childhood physical and emotional neglect and later violent offending has gained more attention over the last years (46). Childhood neglect is indeed the most common form of child maltreatment (46, 47). In line with our results, childhood neglect has been hypothesized to contribute to later violence risk by mediating social information processes including hostile attribution biases (physical neglect) and negative emotional responses (emotional neglect) (48), but these processes can also be affected by childhood emotional abuse. Children who have been exposed to neglect show delayed language development, cognitive deficits, and internalizing and externalizing behavioral problems (49), which may be risk indicators both for schizophrenia and violence. Accordingly, the most frequent subtype of childhood trauma found in a schizophrenia cohort, independent of violence history, was emotional neglect (11). This further corroborates the importance of the subdomain neglect to both schizophrenia and violence risk, in line with the results from our subject sample.

Finally, sexual abuse scores were significantly higher in SCZ-V, but not SCZ-NV compared to HC, and not between the SCZ groups. This is in line with the results from a recent robust clinical study of almost 1500 schizophrenia patients, where childhood sexual abuse emerged as a risk factor for violence (50). Despite the fact that the SCZ-NV group did not differ from HC on sexual abuse scores in our study, previous research in substantially larger cohorts report a 10-fold increase in psychosis risk among persons exposed to childhood sexual abuse (51), and an OR > 2 for psychosis, increasing with age in adolescence, in sexually abused persons (52). As such, the higher sexual abuse scores in the SCZ-V group may reflect increased risk for both violence and psychosis.

### Limitations

The self-report nature of the CTQ is a limitation in this study. The memories of unpleasant events back in time might be suppressed, resulting in biased reporting. Patients could also underestimate history of trauma, hence improving the likelihood of obtaining more false-negatives than false-positives (53). However, these issues are not exclusive to the SCZ-V sample, and Liebschutz et al. have reported that the CTQ does give a reasonably accurate representation of childhood trauma exposure (30). Another limitation could be our use of median values for each of the subdomains in the CTQ. The subdomain scores range from 5 to 25, which represent a great variation in severity. An alternative way to determine severity would be to define a threshold value for each of the subdomains, and to dichotomize the subdomain scores accordingly. A problem with this approach is that heterogeneity and variance in the dataset might disappear (42), and the dichotomization may be somewhat arbitrary even though a key to severity-categorization for the different subdomains has been suggested (54). Finally, the small sample size not only represents the difficulties in conduction research in this patient group, but also affects the robustness of the statistical analyses with a risk of type-II-errors which calls for future replication in larger subject samples.

### Conclusion

SCZ-V patients reported higher exposure to childhood trauma than SCZ-NV, and both groups reported higher exposure than HC. The results point toward childhood physical and emotional neglect to be of specific importance to later violence in schizophrenia, which may be an area for future prevention and clinical attention. To better understand the specific patterns of childhood trauma associated with violence later in life and in order to prevent or treat future violence risk among psychosis patients, replication in larger subject samples are called for.

## Data Availability Statement

The datasets generated for this study will not be made publicly available because we have not received permission from the ethics committee to publish the datasets. Requests to access these datasets should be directed to the corresponding author.

## Ethics Statement

The studies involving human participants were reviewed and approved by Regional Committees for Medical and Health Research Ethics in Norway. The patients/participants provided their written informed consent to participate in this study.

## Author Contributions

GS has taken part in the inclusion of patients, has performed statistical analyses, and has been writing and editing the paper. AJ has assisted in selection of the SCZ-V group and editing of the paper. EB has assisted with statistical analyses and with writing and editing of the paper. NT has wetted the SCZ-NV group to verify that the individuals do not have a history of violence, and she has taken part in editing of the paper. CR has taken part in the inclusion of patients and in the editing of the paper. CF has been advising and taken part in the writing and editing of the paper. OA has been designing the study and taken part in editing of the paper. IM contributed to the statistical analyses, has been designing and supervising the study, and taken part in editing of the paper. UH has been designing and supervising the study, taken part in inclusion of patients and has been writing and editing the paper.

## Funding

This work was supported by the South-Eastern Norway Health Authorities (grant number 2016-044 and 2012-132); Norwegian Research Council (grant number 223273), the KG Jebsen Stiftelsen, and the authors’ institutions. The open access publication is funded by Østfold Hospital Trust.

## Conflict of Interest

The authors declare that the research was conducted in the absence of any commercial or financial relationships that could be construed as a potential conflict of interest.
